# Indoxyl sulfate, a uremic toxin in chronic kidney disease, suppresses both bone formation and bone resorption

**DOI:** 10.1002/2211-5463.12258

**Published:** 2017-07-20

**Authors:** Kenta Watanabe, Tsukasa Tominari, Michiko Hirata, Chiho Matsumoto, Junya Hirata, Gillian Murphy, Hideaki Nagase, Chisato Miyaura, Masaki Inada

**Affiliations:** ^1^ Cooperative Major of Advanced Health Science Tokyo University of Agriculture and Technology Koganei Japan; ^2^ Department of Biotechnology and Life Science Tokyo University of Agriculture and Technology Koganei Japan; ^3^ Institute of Global Innovation Research Tokyo University of Agriculture and Technology Koganei Japan; ^4^ Safety Research Center Kureha Corporation Tokyo Japan; ^5^ Department of Oncology Cancer Research UK Li Ka Shing Centre Cambridge Institute University of Cambridge UK; ^6^ Nuffield Department of Orthopaedics, Rheumatology and Musculoskeletal Sciences Kennedy Institute of Rheumatology University of Oxford UK

**Keywords:** bone formation, bone loss, chronic kidney disease, indoxyl sulfate, osteoblast, osteoclast

## Abstract

Abnormalities of bone turnover are commonly observed in patients with chronic kidney disease (CKD), and the low‐turnover bone disease is considered to be associated with low serum parathyroid hormone (PTH) levels and skeletal resistance to PTH. Indoxyl sulfate (IS) is a representative uremic toxin that accumulates in the blood of patients with CKD. Recently, we have reported that IS exacerbates low bone turnover induced by parathyroidectomy (PTX) in adult rats, and suggested that IS directly induces low bone turnover through the inhibition of bone formation by mechanisms unrelated to skeletal resistance to PTH. To define the direct action of IS in bone turnover, we examined the effects of IS on bone formation and bone resorption *in vitro*. In cultures of mouse primary osteoblasts, IS suppressed the expression of osterix, osteocalcin, and bone morphogenetic protein 2 (BMP2) mRNA and clearly inhibited the formation of mineralized bone nodules. Therefore, IS directly acts on osteoblastic cells to suppress bone formation. On the other hand, IS suppressed interleukin (IL)‐1‐induced osteoclast formation in cocultures of bone marrow cells and osteoblasts, and IL‐1‐induced bone resorption in calvarial organ cultures. In cultures of osteoblasts, IS suppressed the mRNA expression of RANKL, the receptor activator of NF‐κB ligand, which is a pivotal factor for osteoclast differentiation. Moreover, IS acted on osteoclast precursor, bone marrow‐derived macrophages and RAW264.7 cells, and suppressed RANKL‐dependent differentiation into mature osteoclasts. IS may induce low‐turnover bone disease in patients with CKD by its direct action on both osteoblasts and osteoclast precursors to suppress bone formation and bone resorption.

**Abbreviations**
BMM: bone marrow macrophages
BMP2: bone morphogenetic protein 2
CKD: chronic kidney disease
CKD‐MBD: CKD‐related mineral and bone disease
IL: interleukin
OATs: organic anion transporters
PTH: parathyroid hormone
PTX: parathyroidectomy
q‐PCR: quantitative PCR
RANK: receptor activator of NF‐κB
RANKL: receptor activator of NF‐κB ligand
sRANKL: soluble RANKL
TRAP: tartrate‐resistant acid phosphatase

Bone metabolism consists of bone formation induced by osteoblasts and bone resorption regulated by osteoclasts. Osteoblasts are derived from mesenchymal stem cells, and the differentiation of the osteoblast precursors to mature osteoblasts is regulated by various factors and transcription factors such as osteocalcin, bone morphogenetic protein 2 (BMP2), and osterix 1, 2. Mature osteoblasts synthesize calcium phosphate crystals and extracellular matrixes, such as type 1 collagen 3, 4, and deposit these substances as bone tissue. Osteoclast precursors are bone marrow macrophages and express RANK, receptor activator of nuclear factor κB, and the interaction between RANK and RANK ligand (RANKL) expressed on the osteoblast surface induces macrophage differentiation into mature osteoclasts 5, 6. Transcription factors such as NFATc1 are involved in the process of osteoclast differentiation 7. It is well known that osteoblasts and osteoclasts cooperatively regulate bone turnover, and this communication is called bone coupling 8.

Abnormalities of bone turnover are commonly observed in patients with chronic kidney disease (CKD), and this was recently termed CKD‐related mineral and bone disease (CKD‐MBD) 9. The CKD‐MBD Work Group reported that various bone abnormalities, including osteitis fibrosa, adynamic bone disease, and osteomalacia, occurred in patients with CKD stage 3–5 (83%) and with dialysis (98%) 10. It is known that the risk of hip fractures increased in dialysis patients compared to the general population 11, and mortality associated with hip fracture in patients with hemodialysis was higher than that in fracture‐free patients with hemodialysis 12. Thus, abnormalities of bone turnover are thought to be associated with risk of fracture and mortality in patients with CKD.

Renal dysfunction leads to the accumulation of uremic retention solutes in patients with CKD 13, 14, and the European Uremic Toxin Work Group proposed more than 100 substances which are classified as uremic toxins by the molecular weight and the ability of protein binding 13, 15. Some uremic toxins show adverse biological impacts for the cardiovascular system in patients with CKD and CKD model animals 15, 16. Indoxyl sulfate (IS) is an organic anion uremic toxin and is known as a representative toxin in patients with CKD 13. Dietary tryptophan can be metabolized to indole by intestinal bacteria, and absorbed indole is transported to the liver where it is converted to IS 17, Fig. 1. IS is rapidly excreted into urine in healthy subjects, but it accumulates in the blood of patients with impaired renal function, and is involved in glomerular sclerosis and renal fibrosis by the progression of CKD in rats 18, 19.

**Figure 1:**
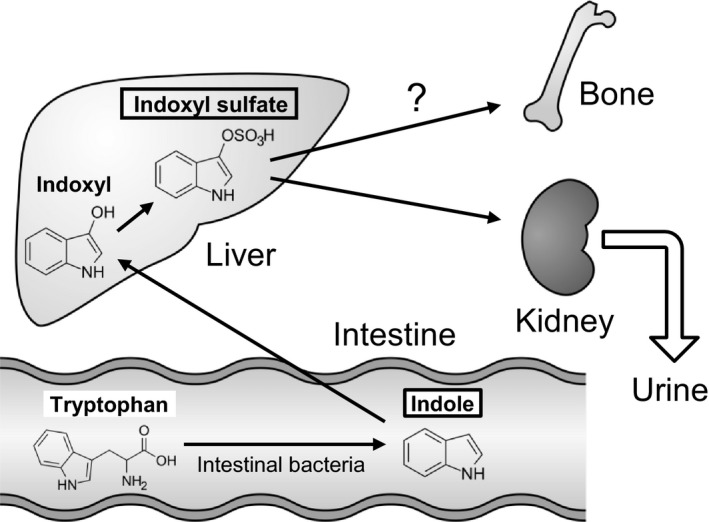
Schematic drawing of the metabolic pathway for the synthesis of indoxyl sulfate (IS) and possible action in bone tissues.

Low‐turnover bone disease is commonly observed in patients with CKD, which was associated with low serum parathyroid hormone (PTH) level and skeletal resistance to PTH 20, 21. We have recently reported that IS exacerbates low bone turnover induced by parathyroidectomy (PTX) in adult rats, suggesting that IS directly inhibited bone formation by mechanisms unrelated to skeletal resistance to PTH 22. In this study, we examined the effects of IS on bone formation and bone resorption in cultures, and suggested the direct action of IS in bone tissues with low turnover in animals and patients with CKD.

## Materials and methods

### Animals and reagents

Newborn and six‐week‐old *ddy* mice were obtained from Japan SLC Inc. (Shizuoka, Japan). All procedures were performed in accordance with the institutional guidelines for animal research, and the experimental protocol was approved by the Animal Care and Use Committee of the Tokyo University of Agriculture and Technology. IL‐1 was obtained from R&D Inc. Soluble RANKL (sRANKL) was obtained from Peprotech Co. Ltd. IS was obtained from Glycosynth, Co. Ltd., Cheshire, UK.

### Culture of primary mouse osteoblastic cells

Primary osteoblastic cells were isolated from newborn mouse calvariae after five routine sequential digestions with 0.1% collagenase and 0.2% dispase, as described previously 23. Osteoblastic cells were collected from fractions 2‐4 and combined, and cultured for 3 days in αMEM with 10% FBS under 5% CO_2_ in air at 37 °C. After the cells reached to confluence, they were trypsinized, counted, and used for the respective experiment.

### Bone formation with mineralization

Primary osteoblastic cells were cultured for 14 days in a medium containing bone‐inducing factors, ascorbic acid (50 μg·mL^−1^), and β‐glycerophosphate (10 mM), to form calcified bone nodules. For the control culture, a medium without bone‐inducing factors was used. IS was added to the medium containing bone‐inducing factors. After culturing, the areas of alizarin‐positive cells were defined as mineralized bone nodules. The areas of alizarin‐positive bone nodules were measured on NIH images.

### Bone‐resorbing activity in the organ cultures of mouse calvaria

Calvariae were collected from newborn mice, dissected in half, and cultured for 24 h in BGJb containing 1 mg·mL^−1^ of bovine serum albumin. After 24 h, the calvariae were transferred into a new medium with or without IL‐1 and IS, and cultured for 5 days. The bone‐resorbing activity was expressed as the increase in the level of calcium in the medium 23.

### Osteoclast formation in cocultures of mouse bone marrow cells and osteoblasts

Bone marrow cells (3 × 10^6^ cells) were isolated from six‐week‐old mice and cocultured with the primary osteoblastic cells (1 × 10^4^ cells) in αMEM containing 10% FBS 23. After culturing for 7 days, the cells adhering to the well surface were stained for tartrate‐resistant acid phosphatase (TRAP). The TRAP‐positive multinucleated cells that contained three or more nuclei per cell were counted as osteoclasts.

### Osteoclast differentiation from macrophages

Bone marrow macrophages were prepared by 3 days of culturing with M‐CSF and 5 days of culturing with or without soluble RANKL (sRANKL). RAW264.7 cells (a murine macrophage cell line) were also cultured for 5 days with or without sRANKL. The TRAP‐positive multinucleated cells that contained three or more nuclei per cell were counted as osteoclasts.

### Quantitative PCR and RT‐PCR

Total RNA was extracted from mouse osteoblasts and from RAW264.7 cells using ISOGEN (Nippon Gene, Tokyo, Japan). cDNA was synthesized from 5 μg of total RNA by reverse transcriptase (Superscript II Preamplification System, Invitrogen, Carlsbad, CA). The quantitative PCR (q‐PCR) was performed with iQ SYBR Green Supermix (Bio‐Rad). The primers used for the q‐PCR for the mouse RANKL, osterix, Col1a1, osteocalcin, BMP2, Nfatc1, RANK, and TRAP genes were constructed from the sequence of respective gene.

The RT‐PCR was performed to examine the mRNA expression of organic anion transporters (OATs), OAT1 and OAT3, in mouse osteoblasts, bone marrow macrophages, and RAW264.7 cells. The primer pairs used in the RT‐PCR for mouse OAT1, OAT3, and β‐actin genes were constructed from the sequence of respective gene. The PCR product was run on a 1.5% agarose gel and stained with ethidium bromide.

### Statistical analysis

The data are expressed as the means ± SEM. Data were analyzed using one‐way ANOVA, followed by Tukey's test for *post hoc* analysis. Statistical analyses were performed using IBM SPSS Statistics version 23 software.

## Results

### IS suppressed bone formation in osteoblast cultures

**Figure 2:**
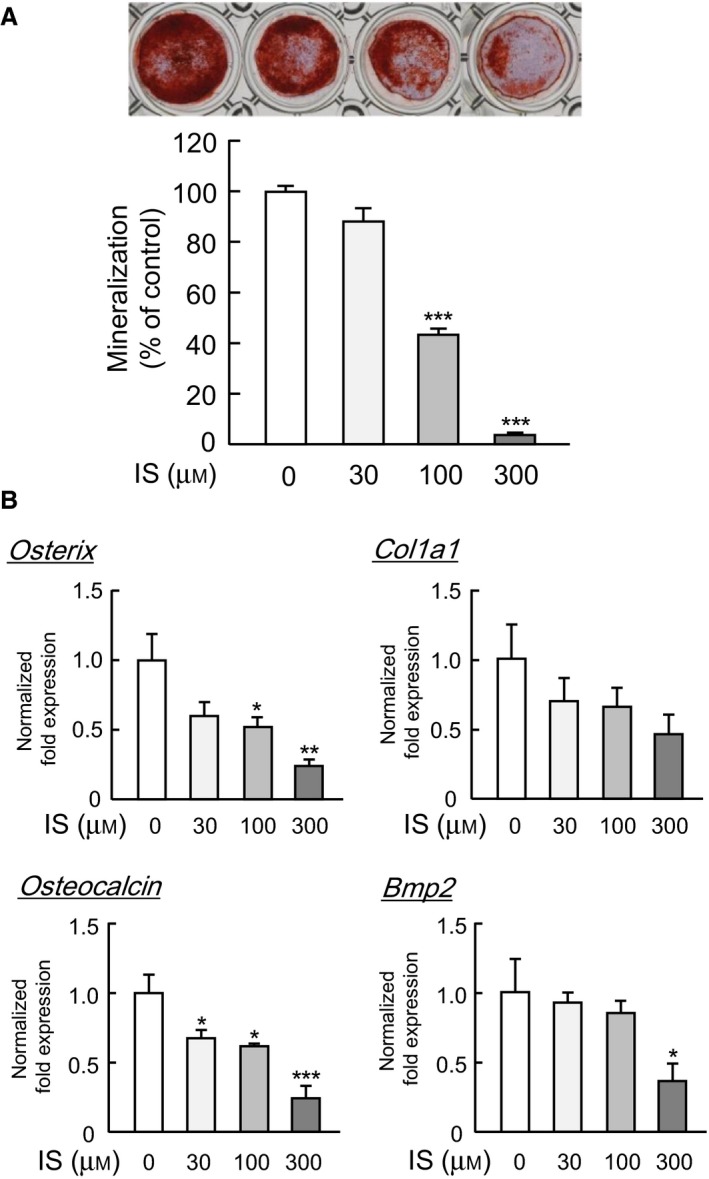
The effects of IS on bone formation and the expression of bone formation‐related genes in osteoblasts. (A) To examine the effects of IS on bone formation *in vitro*, primary osteoblastic cells were cultured with bone‐inducing factors (50 μg/mL of ascorbic acid and 10 mM β‐glycerophosphate) in the presence of IS (30, 100, or 300 μm) for 14 days. The area of alizarin‐positive cells was measured on NIH images. (B) Primary osteoblastic cells were cultured for 14 days with bone‐inducing factors in the presence of IS (30, 100, or 300 μm), and the mRNA expression of osterix, Col1a1, osteocalcin, and BMP2 was measured by a q‐PCR. The data are expressed as the means ± SEM of four independent wells. Asterisks indicate a significant difference: *P < 0.05, **P < 0.01, ***P < 0.001 vs. control.

We first examined the effects of IS on bone formation in primary osteoblast cultures. When osteoblasts were cultured with a medium containing bone‐inducing factors, ascorbic acid, and β‐glycerophosphate, alizarin‐stained mineralized bone nodules could be detected on day 14 (Fig. 2A). The addition of IS, 30‐300 μm, suppressed the formation of mineralized bone nodules in the cultures of primary mouse osteoblasts in a dose‐dependent manner (Fig. 2A). The expression of bone formation‐related gene such as osterix, osteocalcin, and BMP2 mRNA in osteoblasts was found to be suppressed by the addition of IS (Fig. 2B). The mRNA expression of collagen, col1a1, tended to be suppressed by IS, but was not significant. Osterix, osteocalcin, and BMP2 are all important for osteoblast differentiation into mature osteoblasts. Thus, IS directly acted on osteoblasts to suppress bone formation by inhibiting the expression of bone formation‐related genes.

### Effects of IS on IL‐1‐induced osteoclastic bone resorption

**Figure 3:**
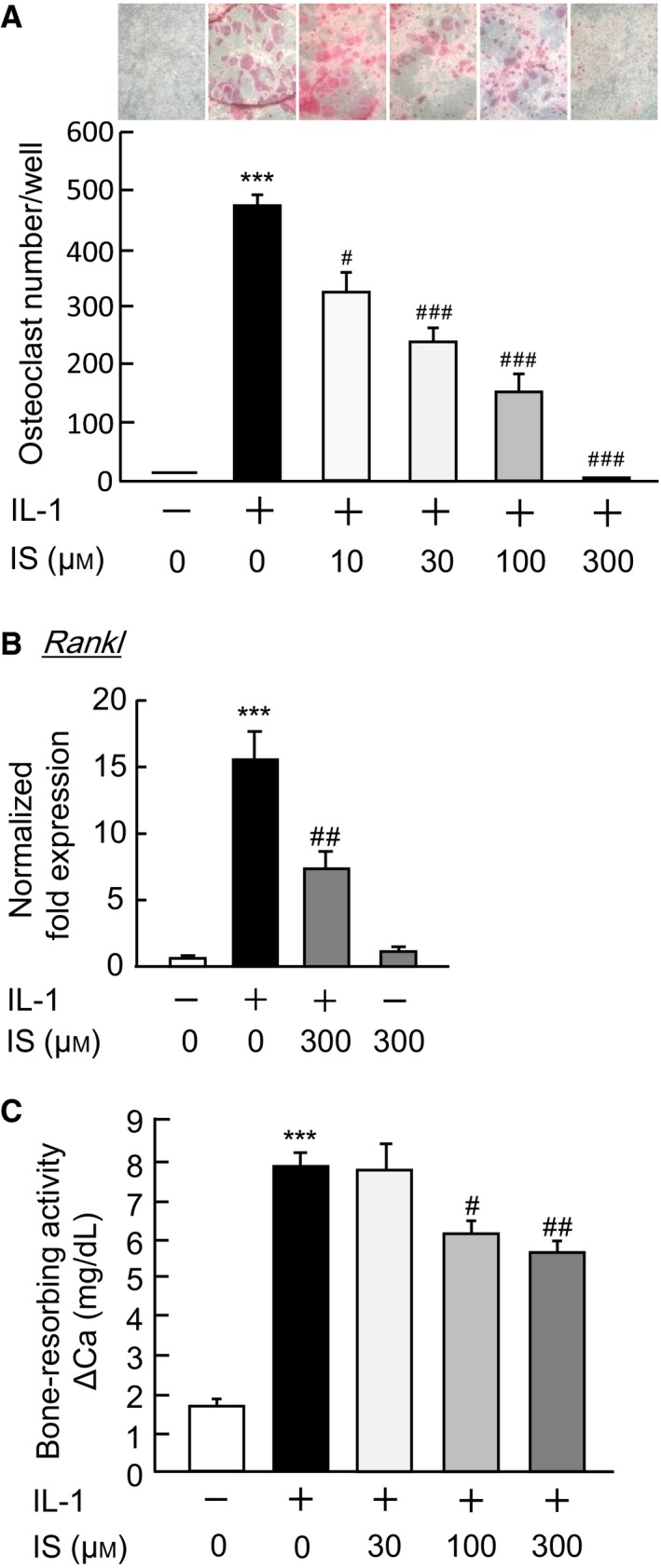
The effects of IS on osteoclast formation and bone resorption. (A) Mouse bone marrow cells and osteoblastic cells were cocultured for 7 days in the presence of IL‐1 (2 ng·mL^−1^) with and without IS (10, 30, 100, or 300 μm). The cells were stained for TRAP to detect osteoclasts. (B) Mouse osteoblasts were treated with IL‐1 (2 ng·mL^−1^) with or without IS (300 μm) for 24 h, and the total RNA was extracted. The mRNA expression of RANKL was detected by a q‐PCR. (C) Mouse calvariae were cultured for 24 h in BGJb medium containing 1 mg·mL^−1^ of BSA, and transferred to new media to culture for 5 days, with or without IL‐1 (2 ng·mL^−1^) and with or without IS (30, 100, or 300 μm). The concentration of calcium in the medium was measured to calculate the bone‐resorbing activity. The data are expressed as the means ± SEM of three to four independent wells. Asterisks and hashes indicate a significant difference: ***P < 0.001 vs. control, ^*#*^ *P* < 0.05, ^*##*^ *P* < 0.01, ^*###*^ *P* < 0.001 vs. IL‐1.

In the cocultures of mouse bone marrow cells and osteoblasts, IL‐1 markedly induced the formation of TRAP‐positive osteoclasts, while the addition of IS suppressed IL‐1‐induced osteoclast formation in a dose‐dependent manner (Fig. 3A). It is well known that bone‐resorbing factors such as IL‐1 induce RANKL expression in osteoblasts to elicit osteoclast differentiation. We therefore examined the effects of IS on the expression of RANKL in osteoblasts. In the cultures of primary mouse osteoblasts, the addition of IS suppressed the mRNA expression of RANKL that was induced by IL‐1 in a q‐PCR assay (Fig. 3B). These results indicate that IS acts on osteoblasts to suppress the expression of RANKL and to negatively regulate IL‐1‐induced osteoclast formation.

A mouse calvarial organ culture is a typical *ex vivo* assay system for defining the effects of a test compound on bone resorption and bone loss. Bone‐resorbing factors such as IL‐1 are known to induce bone resorption in this model. Using *ex vivo* cultures, we examined the effects of IS on IL‐1‐induced bone‐resorbing activity. The application of IL‐1 markedly induced bone‐resorbing activity, while IS (30–300 μm) significantly suppressed bone resorption in a concentration‐dependent manner (Fig. 3C).

### IS suppressed the differentiation of macrophages into mature osteoclasts

**Figure 4:**
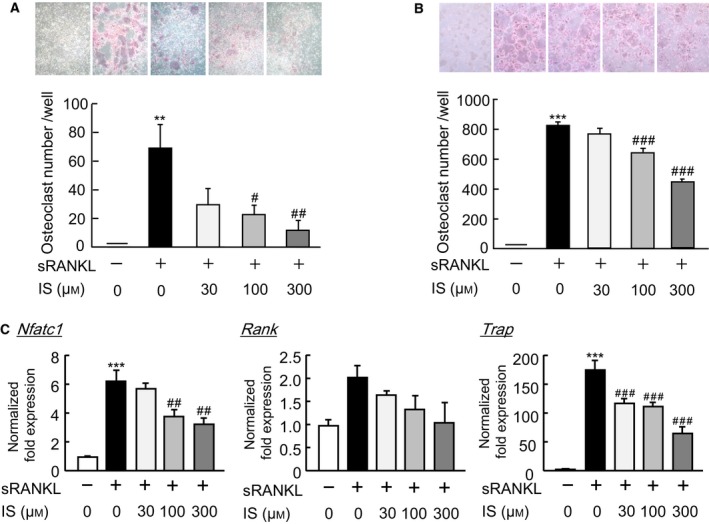
IS acts on macrophages and suppresses RANKL‐dependent osteoclast formation. (A) Bone marrow cells were collected from mice and cultured for 5 days in the presence of M‐CSF. Cells were cultured for three additional days in the presence of M‐CSF and sRANKL (100 ng·mL^−1^) with and without IS (30, 100, or 300 μm). (B) RAW264.7 cells (a murine macrophage cell line) were cultured for 5 days with IS (30, 100, or 300 μm) in the presence of sRANKL (100 ng·mL^−1^). (C) RAW264.7 cells were cultured for 5 days with IS (30, 100, or 300 μm) in the presence of sRANKL (100 ng·mL^−1^), and the mRNA expression of Nfatc1, Rank, and TRAP was measured by a q‐PCR. The data are expressed as the means ± SEM of three to five independent wells. Asterisks and hashes indicate a significant difference: **P < 0.01, ***P < 0.001 vs. control, ^*#*^ *P* < 0.05, ^*##*^ *P* < 0.01, ^*###*^ *P* < 0.001 vs. sRANKL.

Bone marrow macrophages, which are precursor cells for osteoclasts, can differentiate into mature osteoclasts by RANK/RANKL‐mediated mechanisms. To examine the possible actions of IS in relation to the osteoclast precursors, IS was added to the cultures of bone marrow macrophages and RAW264.7 cells (a mouse macrophage cell line) in the presence of sRANKL. The differentiation of bone marrow‐derived macrophages induced by M‐CSF into mature osteoclasts was clearly suppressed by adding IS (Fig. 4A). IS dose dependently suppressed the sRANKL‐dependent differentiation of RAW264.7 cells into osteoclasts (Fig. 4B). In RAW264.7 cells, sRANKL markedly induced the expression of Nfatc1, Rank, and TRAP mRNA for the differentiation into mature osteoclasts, and IS significantly suppressed the expression of these genes (Fig. 4C). As Nfatc1 is an essential transcription factor for osteoclast differentiation, IS may act on macrophages to inhibit their differentiation into osteoclasts.

### Expression of OAT3 in osteoblasts, macrophages, and RAW264.7 cells

**Figure 5:**
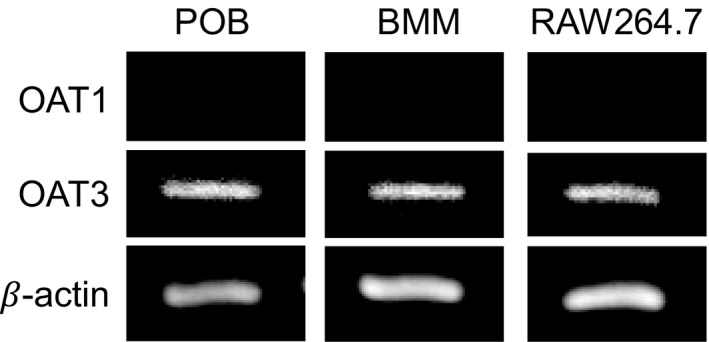
Expression of OAT3 mRNA in osteoblasts, bone marrow macrophages, and RAW264.7 cells. Total RNA was extracted from mouse primary osteoblasts (POB), bone marrow macrophages (BMM), and RAW264.7 cells, and the expression of OAT1 and OAT3 was examined by RT‐PCR.

OATs are known to eliminate organic anions from cells. In kidney, OAT1 and OAT3 localized in the proximal tubules and involved in uptake of organic anions from the blood 24. To discuss the possible roles of OAT1 and OAT3 in bone tissues, we examined the mRNA expression of these OATs in osteoblasts using RT‐PCR and found that osteoblasts expressed only OAT3, but not OAT1 (Fig. 5). In addition, bone marrow macrophages and RAW264.7 cells also expressed OAT3 mRNA, but not OAT1 (Fig. 5). These data suggest that OAT3 may be involved in the mechanism of IS action in bone tissues.

## Discussion

Using CKD rat models, previous studies have indicated that an increase in IS in the blood is related to glomerular sclerosis, renal fibrosis, and the progression of CKD in rats 18, 21. IS is known to induce proximal tubular injury via the increase in free radial production 25, cardiovascular disease 26, 27, and renal anemia 28, 29. Previous studies have suggested that the low‐turnover bone loss is associated with low serum PTH level and with skeletal resistance to PTH in patients with CKD 20, 21. Nii‐Kono *et al*. 30 have shown that IS suppresses PTH‐stimulated intracellular cAMP production, PTH receptor expression, and induces oxidative stress in primary cultured murine osteoblastic cells. However, we have recently reported that IS exacerbates low bone turnover through using PTX rats, suggesting that IS‐induced low bone turnover may be due to the inhibition of bone formation by mechanisms unrelated to skeletal resistance to PTH 22. Oral administration of indole induced low bone turnover in PTX rats, and the serum IS levels in indole‐treated rats on weeks 2 and 4 were 5.1 mg·dL^−1^ (240 μm) and 3.9 mg·dL^−1^ (183 μm), respectively 22. Niwa *et al*. 18 reported that serum IS level was 1.8 ± 1.5 mg·dL^−1^ (84 μm) in predialysis CKD patients, and 5.3 ± 2.1 mg·dL^−1^ (249 μm) in patients on hemodialysis before dialysis. In the present study, we have found that IS, 100–300 μm, acted on osteoblasts and suppressed bone formation in osteoblast cultures. The concentrations of IS used in the present study are similar to serum IS levels in indole‐treated rats and in patients with CKD, suggesting that the direct action of IS in bone formation may be occurred *in vivo* in CKD animals and patients.

Various uremic toxins accumulate in blood during renal failure, and these toxins are classified by molecular weight and mode of protein binding. In addition to IS, p‐cresyl sulfate (PCS) is a protein‐bound uremic toxin, and serum PCS levels are significantly higher in patients with CKD compared with those of the healthy control 31. Tanaka *et al*. 32 have reported that PCS induces osteoblast dysfunction by the suppression of PTH‐induced cAMP production and by the induction of intracellular production of reactive oxygen species. Further *in vivo* studies are needed to define the roles of PCS in bone metabolism.

In the present study, IS acted on osteoblasts and suppressed bone formation with mineralization by inhibiting the mRNA expression of osterix, osteocalcin, and BMP2. However, the molecular mechanisms of IS action in osteoblasts are unknown. The OAT family consists of six isoforms, and all OAT are expressed in kidney, while some OAT are expressed in liver, brain, and placenta 33. In the CKD rats 34 and patients with CKD 35, IS may induce the nephrotoxicity by the mechanisms involving OAT1 and OAT3. In the present study, we detected the mRNA expression of OAT3, but not OAT1, in osteoblasts, bone marrow macrophages, and RAW264.7 cells (Fig. 5). Although further studies are needed to define the roles of OATs in IS action in osteoblasts and osteoclast precursors, OAT3 may be involved in the mechanism of IS action in bone. After the uptake of IS into the target cells, IS may modulate some signal transductions in osteoblasts and macrophages. Tanaka *et al*. 32 have reported that IS induces ERK1/2 phosphorylation in mouse osteoblasts, but PCS enhances JNK and p38 MAPK pathways in osteoblasts. Further studies using uremic toxins and OATs are needed to unveil the pathogenesis of CKD.

Roles of inflammatory cytokines in the pathogenesis of CKD have been reported, and increased levels of serum cytokines such as IL‐1, IL‐6, and TNF‐α were associated with poor clinical outcomes in patients with CKD 36. In bone tissues, IL‐1 is a typical bone‐resorbing cytokine associated with inflammation, but other cytokines such as TNF‐α, IL‐6, and IL‐17 are known to be involved in bone destruction associated with rheumatoid arthritis. Therefore, we used IL‐1, a general bone‐resorbing cytokine, in the present study to examine the effects of IS on bone resorption *in vitro*. In the present study, IS suppressed IL‐1‐induced osteoclast formation in the cocultures of bone marrow cells and osteoblasts, and also suppressed IL‐1‐induced bone resorption in calvarial organ cultures. As IS acted on osteoblasts to suppress the expression of RANKL, IS may suppress bone resorption by the mechanisms involving osteoblasts. In addition, IS acted on osteoclast precursors to suppress the RANKL‐dependent differentiation into mature osteoclasts. These results are consistent with the data reported by Mozar *et al*. 37 using RAW264.7 cells. Based on these results, IS may act on both osteoblasts and osteoclast precursors to suppress osteoclast differentiation and bone resorption. In our previous study using rats, the treatment with indole slightly suppressed bone resorption, measured by bone morphometry markers such as OcS/BS and ES/BS, although there were no significant differences 22. Therefore, further studies are needed to define the roles of bone resorption regulated by IS in the low‐turnover bone disease in patients with CKD.

In conclusion, the present study demonstrated for the first time that IS directly suppresses bone formation and osteoblast/osteoclast coupling *in vitro*. Taken together with our previous report, we suggest here that the exacerbation of low bone turnover by IS may be due to the direct action of IS in bone tissues.

## Author contributions

KW and MI supervised the experiments; KW, TT, MH, and CM designed the experiments and analyzed the data; MI, CM, and JH conceived the project and wrote the article with contributions of all authors; GM and HN provided comments pertaining to the manuscript; and MI and CM supervised and complemented the writing.
